# A Bifunctional Peptide Conjugate That Controls Infections of *Erwinia amylovora* in Pear Plants

**DOI:** 10.3390/molecules26113426

**Published:** 2021-06-05

**Authors:** Pau Caravaca-Fuentes, Cristina Camó, Àngel Oliveras, Aina Baró, Jesús Francés, Esther Badosa, Marta Planas, Lidia Feliu, Emilio Montesinos, Anna Bonaterra

**Affiliations:** 1 LIPPSO, Department of Chemistry, Campus Montilivi, University of Girona, 17003 Girona, Spain; pau.caravaca@udg.edu (P.C.-F.); criscamo18@hotmail.com (C.C.); a.oliveras.rovira@gmail.com (À.O.); marta.planas@udg.edu (M.P.); lidia.feliu@udg.edu (L.F.); 2 Laboratory of Plant Pathology, Institute of Food and Agricultural Technology-CIDSAV-XaRTA, Campus Montilivi, University of Girona, 17003 Girona, Spain; aina.baro@udg.edu (A.B.); jesus.frances@udg.edu (J.F.); esther.badosa@udg.edu (E.B.); emilio.montesinos@udg.edu (E.M.)

**Keywords:** fire blight, antimicrobial peptides, plant-defense elicitors, peptide conjugate

## Abstract

In this paper, peptide conjugates were designed and synthesized by incorporating the antimicrobial undecapeptide **BP16** at the C- or N-terminus of the plant defense elicitor peptide **flg15**, leading to **BP358** and **BP359**, respectively. The evaluation of their in vitro activity against six plant pathogenic bacteria revealed that **BP358** displayed MIC values between 1.6 and 12.5 μM, being more active than **flg15**, **BP16**, **BP359**, and an equimolar mixture of **BP16** and **flg15**. Moreover, **BP358** was neither hemolytic nor toxic to tobacco leaves. **BP358** triggered the overexpression of 6 out of the 11 plant defense-related genes tested. Interestingly, **BP358** inhibited *Erwinia amylovora* infections in pear plants, showing slightly higher efficacy than the mixture of **BP16** and **flg15**, and both treatments were as effective as the antibiotic kasugamycin. Thus, the bifunctional peptide conjugate **BP358** is a promising agent to control fire blight and possibly other plant bacterial diseases.

## 1. Introduction

Fire blight caused by *Erwinia amylovora* is one of the main diseases that affect plants of the family Rosaceae, causing substantial production losses worldwide in important fruit crops, such as apple and pear trees [1,2,3,4]. Management of fire blight relies on a combination of strategies that include cultural practices and/or the use of tolerant cultivars and of preventive bactericide sprays [3]. Preventive applications of bactericides, such as antibiotics and copper compounds, for the control of fire blight, have several drawbacks. In particular, repeated antibiotic treatments have led to the emergence of resistance within the pathogen population which limits their efficacy, and the use of antibiotics is not authorized in many countries [5,6,7,8,9,10]. In the case of copper compounds, they are not always effective, even after multiple applications, and they may cause phytotoxicity by increasing fruit russeting [8]. These limitations entail an impending need to find safer and more effective methods to combat fire blight.

Antimicrobial peptides, found in the defense mechanisms of most living organisms, have been reported as promising compounds for fire blight management [11,12,13,14,15]. These peptides are cationic sequences of 2 to 50 amino acids, able to adopt an amphipathic structure [16,17,18]. In addition to complying with safety standards, they have some advantages over other treatments. In particular, they are biodegradable, and it is difficult for bacteria to develop resistance to them because their mechanism of action mainly involves the disruption of the bacterial cell membrane and, moreover, they are selective for bacterial cells over plant or mammal tissues [16,17,19,20,21].

Plant defense inducers are also viewed as an interesting complement to strategies using bactericides in plant protection. Plants have an immune system able to activate defense mechanisms by innate immunity, and systemic induced resistance (ISR), or/and systemic acquired resistance (SAR) [22,23]. The recognition of different molecular patterns leads to the activation of transcriptional regulators and defense-related genes. Microbe-associated molecular patterns (MAMPs) are able to cause defense priming that triggers the ISR mediated by jasmonic acid and ethylene [23]. Other molecular patterns such as pathogen or damage-associated (PAMPs or DAMPs) induce the pattern-triggered immunity (PTI) followed, in a second phase, by the effector-triggered immunity (ETI). The initiation of ETI results in a local resistance reaction of the infected tissue represented by localized programmed cell death (PCD) called the hypersensitive response (HR) and, subsequently, induces SAR mediated by salicylic acid. Compounds, such as bion, acibenzolar-*S*-methyl, or harpins, have been described as acting as defense elicitors in apple or pear plants, being effective in the control of fire blight [3,24]. Moreover, peptides **flg22** or **flg15**, derived from bacterial flagellin, have been reported to elicit immune responses in a broad variety of plants, including tomatoes or apples [25,26]. Therefore, they could be useful as agents to control fire blight.

The conjugation of two different peptides constitutes an efficient approach to obtain novel compounds with improved antimicrobial activity compared to the individual sequences [27,28,29]. In fact, the conjugation of sequences with antimicrobial activity has been extensively reported [30,31,32,33,34,35]. In this context, we conjugated the antimicrobial peptide **BP100** with fragments of natural antimicrobial peptides to obtain peptide conjugates with improved biological activity against plant pathogens than their corresponding monomers [13]. By contrast, peptide conjugates resulting from the combination of a peptide with antimicrobial activity with another peptide with a different activity are limited [36,37,38]. To the best of our knowledge, the conjugation of an antimicrobial peptide and a plant defense elicitor sequence has only been described by the group of G.-Y. Chen, who conjugated the active domains of cecropin A and melittin to the elicitor harpin Hpa1 to obtain a chimeric protein that conserved the biological activities of the parent monomers [39]. In addition, few papers describing the design of new peptide conjugates have evaluated the effect on the activity of the order of the monomers in the sequence of these conjugates [13,40,41,42,43]. Results from this evaluation point out that the biological activity of the peptide conjugates is significantly sensitive to this structural modification.

Studies on antimicrobial peptides are almost solely focused on the use of individual peptides. However, another attractive approach relies on mixtures of different peptides or mixtures of peptides and conventional antibiotics [18,44]. Concerning the combination of two antimicrobial peptides, these mixtures have been reported to cause a higher antimicrobial effect than the individual components [18,45,46,47,48]. In this context, the differences in biological activity between the conjugation of two peptides in a single sequence and a mixture of the two individual peptides have been scarcely analyzed [43,49,50,51,52]. In one of these studies, it was concluded that a hybrid peptide derived from magainin 2 and PGLa showed similar or even stronger antimicrobial activity than an equimolar mixture of magainin and PGLa [49]. Similarly, the hybrid antimicrobial peptide LFchimera, incorporating the sequences of bovine lactoferrampin (265–284) and lactoferricin (17–30), exhibited significantly higher antimicrobial and candidacidal activity than the individual peptides or an equimolar mixture of both of them [51,52]. Recently, Wade et al. demonstrated that peptide conjugates derived from parasin were more active than cocktails of the corresponding individual sequences, while hybrid peptides derived from magainin 2 were at least as active as the mixtures of the corresponding monomers [43].

The aim of the present study was to obtain efficient agents for the management of fire blight by conjugating the antimicrobial peptide **BP16** at the N- or at the C-terminus of the plant defense elicitor **flg15**. The linear undecapeptide **BP16** was previously identified to display moderate antimicrobial activity against *E. amylovora* with low toxicity [2,11,20]. We evaluated the resulting peptide conjugates to determine the antimicrobial activity against several bacterial phytopathogens. Furthermore, the capacity of the best peptide conjugate to induce the expression of defense-related genes on tomato plants was studied. Finally, the effectiveness of this peptide conjugate was assessed in whole plants against fire blight disease in controlled greenhouse environmental conditions. The activity of the peptide conjugates was compared to that of a mixture of both monomers **BP16** and **flg15**.

## 2. Results

### 2.1. Design and Solid-Phase Synthesis of the Peptides

Peptide conjugates were designed by combining the antimicrobial peptide **BP16** and the plant defense elicitor peptide **flg15** (Table 1). With the aim of studying the influence of the order of the monomers on the biological activity, **BP16** was conjugated at the C- or at the N-terminus of **flg15**, resulting in peptide conjugates **BP358** (flg15-BP16) and **BP359** (BP16-flg15), respectively.

The synthesis was performed in solid-phase following a standard 9-fluorenylmethoxycarbonyl (Fmoc)/*tert*-butyl (^t^Bu) strategy to yield a C-terminal amide for **BP16** and **BP358** and a C-terminal carboxylic acid in the case of **flg15** and **BP359**. After purification, peptides were obtained at >99% HPLC purity and were characterized by HRMS (Table 1) (Supplementary Materials for characterization of **BP16**, **flg15**, **BP358** and **BP359**).

### 2.2. In Vitro Biological Activity of Peptides

The antibacterial activity of the peptide conjugates **BP358** and **BP359** was assayed against *E. amylovora*, *Pseudomonas syringae* pv. syringae, *Pseudomonas syringae* pv. actinidiae, *Xanthomonas arboricola* pv. pruni, *Xanthomonas fragariae*, and *Xanthomonas axonopodis* pv. vesicatoria at 1.6, 3.1, 6.2, 12.5, 25, 50, and 100 μM (Table 2). The monomers **BP16** and **flg15** were included for comparison purposes. In order to analyze the effect of the conjugation, assays were also performed using an equimolar mixture of these two peptides.

**BP16** was active against the above bacteria with MIC values ranging from 3.1 to 50 μM. In contrast, as expected, **flg15** did not display antibacterial activity (MIC > 100 μM). The peptide conjugate **BP358** (flg15-BP16) was considerably more active (MIC values between 1.6 and 12.5 μM) than both **BP359** (BP16-flg15, MIC of 3.1 to > 50 μM) and **BP16** (MIC of 3.1 to 50 μM). In particular, **BP358** showed lower MIC values than **BP16** against *E. amylovora*, *X. arboricola* pv. pruni, *X. fragariae*, and *X. axonopodis* pv. vesicatoria. The mixture of the monomers **BP16** and **flg15** exhibited similar antibacterial activity to that of **BP16** (MIC of 3.1 to 50 μM). In addition, the reference antibiotic kanamycin sulfate displayed a similar MIC than the peptides against *E. amylovora* (MIC of 6.2 to 12.5 μM).

**Figure 1:**
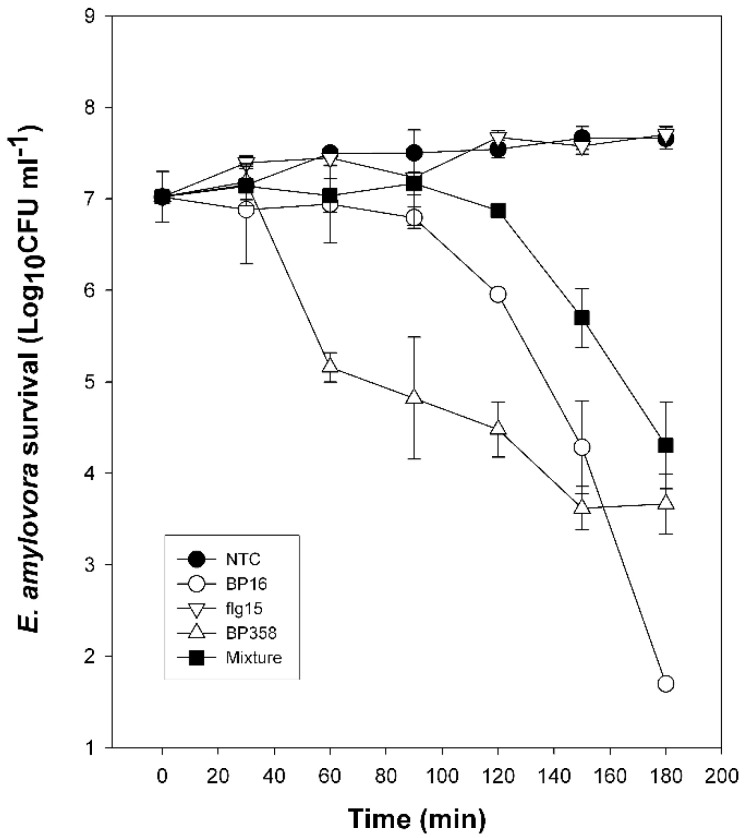
Kinetics of survival of *E. amylovora* in the presence of peptides. Bacterial suspensions were untreated (NTC) or treated with **BP16** (50 µM), **flg15** (100 µM), **BP358** (6.2 µM), or a mixture of **flg15** and **BP16** at equal molar quantities (25 μM) (Mixture). Viable cells were determined at different time intervals.

Bactericidal activity was evaluated by comparing the time course to kill mid-logarithmic-phase culture suspensions of *E. amylovora*. As shown in Figure 1, at the concentration tested, **BP358**, **BP16,** and the mixture of **BP16** and **flg15** showed a bactericidal effect. They reduced *E. amylovora* population 3–4 log after a 180 min exposure period; **BP358** was the peptide that showed faster bactericidal activity.

The toxicity of these peptides to eukaryotic cells was determined as the ability to lyse erythrocytes in comparison to melittin (100% hemolysis). All the peptides, as well as the mixture of **BP16** and **flg15**, displayed low hemolytic activity with percentages of hemolysis ranging from 0% to 5% at 375 μM (Table 2). These peptides were also evaluated for their effect on tobacco leaves. Melittin was used as a positive control, which caused a brown necrotic area of around 2 cm diameter at 250 μM. In contrast, the monomers (**BP16** and **flg15**) and the conjugates (**BP358** and **BP359**) had a considerably less toxic effect at this concentration, causing a lesion diameter of 0.26, 0, 0.42, and 0.30 cm, respectively. The phytotoxicity of the mixture of **BP16** and **flg15** was similar to that of the monomers, causing a lesion diameter of 0.30 cm at 250 μM.

### 2.3. Effect of Peptides on Defense Gene Expression of Tomato Plants

The capacity to induce the expression of genes related to defense response in tomato plants was evaluated for the peptide conjugate **BP358**, the corresponding monomers **BP16** and **flg15**, and an equimolar mixture of **BP16** and **flg15** (Table 3). Acibenzolar-*S*-methyl was included as positive control. The relative quantification for the expression of the selected genes was performed using the *ΔΔCt* method [53]. A fold induction above 2 was considered overexpression.

Tobacco plants treated with acibenzolar-*S*-methyl showed overexpression of 9 out of the 11 genes analyzed (*Harp*, *PR1*, *GluA*, *PPO*, *LOX*, *PinII, Sub1*, *ERT3*, and *BCB*). The monomer **flg15** caused overexpression of all tested genes except for *LOX*, whereas **BP16** only induced the overexpression of *Tas14.* Notably, the peptide conjugate **BP358** promoted the overexpression of 6 genes: *Harp*, *GluA*, *Sub1*, and *BCB*, with a relative overexpression of more than 3-fold, and *LOX* and *Osm2* with a relative overexpression between 2- and 3-fold. Concerning the mixture of **BP16** and **flg15**, it caused the overexpression of two genes, *BCB* and *GluA*.

### 2.4. Activity of Peptides in Planta

**Figure 2:**
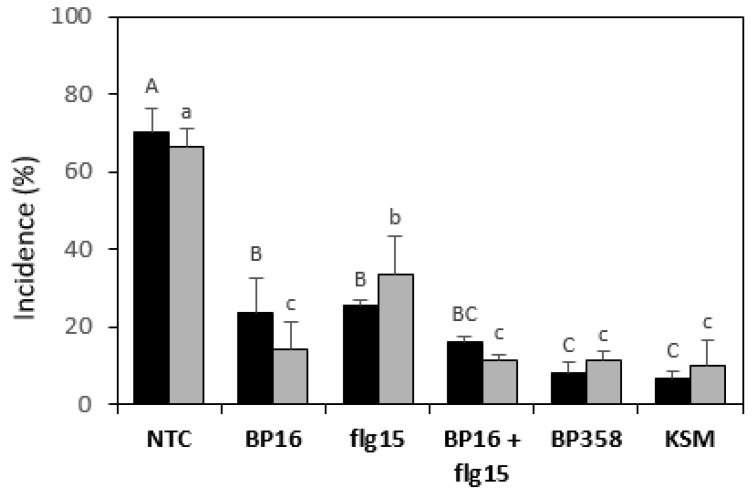
Effect of the peptides on the incidence of *E. amylovora* infections in pear plants. Black bars correspond to assay one and grey bars to assay two. A non-treated control (NTC) and a reference treatment with kasugamycin (KSM) were included. The treatment with a mixture of **BP16** and **flg15** is labeled as **BP16 + flg15**. The confidence intervals for the means are indicated on top of the bars. Different letters (capital letters for assay one and lowercase letters for assay two) show significant differences between the treatments according to Duncan’s test (*p* < 0.05, ANOVA, LSD).

The effect of the peptide conjugate **BP358** to reduce *E. amylovora* infections was tested in potted pear plants assays and compared with that of a mixture containing equal molar quantities of **BP16** and **flg15** (Figure 2). The incidence of the infections was determined. Monomers **BP16** and **flg15** were included for comparison purposes, and water and kasugamycin at 2 g/L, as a non-treated and positive control, respectively. All peptide treatments were performed at 125 µM. Two independent assays were carried out.

Treatment with **BP16** and **flg15** significantly reduced the incidence of fire blight in pear plants compared to the non-treated control. In one of the experiments, the peptide conjugate **BP358** significantly decreased the incidence of the disease compared to the treatment with the monomers **BP16** and **flg15**. In another experiment, **BP358** significantly decreased the incidence compared to **flg15**, but they did not differ from **BP16**. Remarkably, the efficacy of **BP358** did not differ significantly from kasugamycin. Results of the mixture of **BP16** and **flg15** did not differ from those of the monomers in one experiment and were improved compared to **flg15** in the other one. In addition, it should also be pointed out that in both experiments, the efficacy of this mixture did not differ significantly from **BP358** and from kasugamycin.

## 3. Discussion

Conjugation of two peptides to obtain sequences with a better biological profile is a widely used strategy. This approach has led to the design of interesting hybrid peptides with significant antimicrobial activity derived from cecropin A, melittin, lactoferricins, thanatin, cathelicidin, and LL-37, among others [30,31,32,33,34,37,51,52]. Within this field, we described peptide conjugates containing the antimicrobial peptide **BP100** and fragments of cecropin A, melittin, and magainin [13]. These conjugates were more active than the monomers against plant pathogens such as *E. amylovora*, *X. axonopodis* pv. vesicatoria, and *P. syringae* pv. syringae. Interestingly, the hybridization of two peptides with different activity may be a promising approach since it would allow designing bifunctional peptides. In particular, we envisaged that the conjugation of an antimicrobial peptide with a plant defense elicitor peptide would provide sequences with both activities that would be useful for plant protection. Thus, in the present study, peptide conjugates **BP358** (flg15-BP16) and **BP359** (BP16-flg15), resulting from the combination of the antimicrobial peptide **BP16** [11] and the plant defense elicitor peptide **flg15** [25,26,54], were designed and tested against bacterial plant pathogens. In addition, the activity of these two conjugates was compared to that of a mixture of **BP16** and **flg15**.

Peptides **BP16** and **flg15** were poorly active in vitro, and both the conjugation of these sequences and their order in the final peptide conjugate influenced the in vitro antibacterial activity against *E. amylovora*, *P. syringae* pv. actinidiae, *P. syringae* pv. syringae, *X. arboricola* pv. pruni, *X. axonopodis* pv. vesicatoria, and *X. fragariae*. Interestingly, the conjugation of **BP16** and **flg15** led to an enhancement of the activity, in particular, when **BP16** was linked at the C-terminus of **flg15**. The resulting peptide conjugate **BP358** (flg15-BP16) was more active (MIC between 1.6 and 6.2 μM) against *E. amylovora*, *X. arboricola* pv. pruni, *X. axonopodis* pv. vesicatoria, and *X. fragariae* than the antimicrobial peptide **BP16** (MIC between 6.2 and 50 μM), and displayed the same MIC values as **BP16** against *P. syringae* pv. actinidiae and *P. syringae* pv. syringae (MIC between 3.1 and 12.5 μM). In contrast, **BP359**, which included **BP16** at the N-terminus, was only more active than **BP16** against *X. axonopodis* pv. vesicatoria. The difference in activity observed between the two conjugates is in agreement with other studies, which report that the position of the monomers in the final conjugate is crucial for the activity [13,40,41,42,43].

Investigation of antibacterial activity also showed that a mixture containing equal molar quantities of both monomers **BP16** and **flg15** showed similar MIC values as **BP16**, and it was less active than the peptide conjugate **BP358**. This result suggests that these two monomers do not interfere with each other either when mixed or conjugated. Other peptide conjugates with higher activity than that of an equimolar mixture of the corresponding monomers have been described; a hybrid peptide derived from magainin 2 and PGLa [49], the peptide conjugate LFchimera, which incorporates lactoferrampin (265–284) and lactoferricin (17–30) [51,52], and hybrid peptides derived from parisin and BF2 or DesHDAP1 [43].

Concerning the toxicity, the conjugation of **BP16** and **flg15**, the order of the monomers in the resulting peptide conjugates, or their mixture did not influence the hemolysis or the phytotoxicity. In fact, the monomers **BP16** and **flg15**, the peptide conjugates **BP358** and **BP359**, and the mixture of the monomers did not show cytotoxic activity to eukaryotic cells or toxicity to tobacco leaves. According to all these results, the conjugate **BP358** had the best biological profile (MIC values between 1.6 and 12.5 μM and 5% hemolysis at 375 μM) and was selected for further studies.

The elicitation of plant defense was studied in tomato plants using genes related to the salicylic acid, jasmonic acid, or ethylene pathways, as well as with saline stress or wound damage [4,55]. As expected, tomato plants treated with **flg15** overexpressed more genes and more intensely than the reference compound acibenzolar-*S*-methyl. The conjugation of **flg15** with **BP16** resulted in peptide **BP358** that maintained the ability to act as a plant defense elicitor. Interestingly, **BP358** triggered the overexpression of 6 out of 11 genes, sharing with **flg15** the overexpression of genes *Harp*, *GluA*, *Sub1*, *BPB*, and *Osm 2.* The mixture of **flg15** and **BP16** had a lower elicitation activity promoting only the overexpression of genes *GluA* and *BPB.* This result suggests that these two peptides interfere with each other causing a reduction in the plant defense elicitation properties. As well as synergistic effects, antagonistic effects on defense induction have already been observed when using MAMPs mixtures, with a reduction in the reactive oxygen species (ROS) production in *Arabidopsis thaliana* of around 90% [56]. In addition, it cannot be discarded the possibility that **BP16** blocks the binding site of the flagellin receptor FLS2 because it has been reported that other ligands different from flagellin bind to this receptor [57].

In the pear-*E. amylovora* pathosystem plant assays, both the peptide conjugate **BP358** and the mixture of **BP16** and **flg15** are suitable options to reduce the effects of the disease. It is worth mentioning that the monomers displayed higher activity when tested in planta than in vitro. Especially remarkable is the case of **flg15** that was not active in vitro whereas in planta caused a reduction in incidence of 50–64% in comparison to the non-treated control. Although the monomers turned out to be more active, **BP358** and the mixture of **BP16** and **flg15** resulted in even more effective protection of plants from *E. amylovora* infections. **BP358** exhibited a slightly higher efficacy than the mixture, and both were, in general, as effective as the antibiotic kasugamycin. The higher activity of **BP358** could be attributed to its higher plant defense elicitation properties; this fact would also explain the improved activity shown by **flg15** in the assays in planta.

Based on the above considerations, both peptide conjugation and the use of mixtures have shown to be feasible strategies to improve the biological activity of peptides as agents to control plant diseases. On the one hand, the peptide conjugate **BP538** displayed high antimicrobial activity in planta and also promoted the overexpression of a high number of genes related to plant defense response. The use of peptide conjugates in plant protection is advantageous because their production can be achieved using microbial or plant biofactories. On the other hand, the mixture of peptides **flg15** and **BP16** also displayed high activity in planta and, since they are short peptide sequences, their chemical synthesis can be easily performed [13]. Moreover, treatments using peptide mixtures may reduce the development of bacterial resistance. In fact, Rolff et al. demonstrated that the combination of the antimicrobial peptides pexiganan and melittin slowed the evolution of resistance in *Staphylococcus aureus* compared to the use of individual antibiotics or antimicrobial peptides [47,58].

## 4. Materials and Methods

### 4.1. General Methods

Manual solid-phase synthesis was performed in polypropylene syringes (2 or 5 mL) fitted with a polyethylene porous disk. Solvents and soluble reagents were removed by suction. Most chemicals were from commercial suppliers Merck (Madrid, Spain), Iris Biotech GmbH (Marktredwitz, Germany), Carlo Erba (Sabadell, Spain), and used without further purification.

Peptides were analyzed using standard analytical high-performance liquid chromatography (HPLC) conditions with a Dionex liquid chromatography instrument composed of a UV/Vis Dionex UVD170U detector (Thermo Fisher Scientific, Sunnyvale, CA, USA), a P680 Dionex bomb, an ASI-100 Dionex automatic injector, and CHROMELEON 6.60 software (Thermo Fisher Scientific, Sunnyvale, CA, USA). Detection was performed at a wavelength of 220 nm. Solvent A was 0.1% aqueous trifluoroacetic acid (TFA), and solvent B was 0.1% TFA in CH_3_CN. Analyses were carried out with a Kromasil 100 C_18_ (4.6 mm × 40 mm × 3.5 µm) column with a 2–100% B over 7 min at a flow rate of 1 mL/min.

All peptides were purified on a Combi*Flash* Rf200 automated flash chromatography system using Redi*Sep* Rf Gold reversed-phase C_18_ column packed with high-performance C_18_ derivatized silica (Teledyne ISCO, Lincoln, NE, USA).

Electrospray-ionization mass spectrometry (ESI-MS) analyses were performed at the Serveis Tècnics de Recerca of the University of Girona with an Esquire 6000 ESI ion Trap LC/MS (Bruker Daltonics, Billerica, MA, USA) instrument equipped with an electrospray ion source. The instrument was operated in the positive ESI(+) ion mode. Samples (5 µL) were introduced into the mass spectrometer ion source directly through an HPLC autosampler. The mobile phase (80:20 CH_3_CN/H_2_O at a flow rate of 100 µL/min) was delivered by a 1200 Series HPLC pump (Agilent, Santa Clara, CA, USA). Nitrogen was employed as both the drying and nebulizing gas.

High-resolution mass spectrometry (HRMS) data were recorded on a Bruker MicroTof-QIITM instrument (Bruker Daltonics, Billerica, MA, USA) using ESI ionization source at the Serveis Tècnics de Recerca of the University of Girona. Samples were introduced into the mass spectrometer ion source by direct infusion using a syringe pump and were externally calibrated using sodium formate. The instrument was operated in the positive ion mode.

### 4.2. General Procedure for the Solid-Phase Synthesis of Peptides

Peptides were synthesized manually by the solid-phase method using a standard Fmoc/^t^Bu strategy. A Fmoc-Rink-MBHA resin (0.55 mmol/g) was used for the synthesis of **BP16**, a Fmoc-Rink-ChemMatrix resin (0.66 mmol/g) for the synthesis of the conjugate **BP358** (flg15-BP16), and a PAC-ChemMatrix resin (0.66 mmol/g) for the synthesis of **flg15** and the conjugate **BP359** (BP16-flg15) (Company, City, State Abbrev. if USA or Canada, Country). Fmoc-Leu-OH, Fmoc-Lys(Boc)-OH, Fmoc-Phe-OH, Fmoc-Ile-OH, Fmoc-Ala-OH, Fmoc-Gly-OH, Fmoc-Gln(Trt)-OH, Fmoc-Arg(Pmc)-OH, Fmoc-Ser(^t^Bu)-OH, Fmoc-Asp(O^t^Bu)-OH, and Fmoc-Asn(Trt)-OH were used as amino acid derivatives. The coupling of the first amino acid (5 equiv.) onto the PAC-derivatized resins was performed in the presence of *N,N*’-diisopropylcarbodiimide (DIC) (5 equiv.), 4-dimethylaminopyridine (DMAP) (0.5 equiv.), and *N,N*’-diisopropylethylamine (DIEA) (1 equiv.) in *N,N*-dimethylformamide (DMF) at room temperature for 2 h while stirring. This treatment was repeated twice and, then the resin was washed with DMF (6 × 1 min) and CH_2_Cl_2_ (3 × 1 min) and dried with diethyl ether (3 × 2 min). The completion of the coupling was checked using a Fmoc test. Then, the resin was acetylated with acetic anhydride/pyridine/CH_2_Cl_2_ (1.35:1.35:18, 2 × 30 min) followed by washes with CH_2_Cl_2_ (3 × 2 min), DMF (3 × 2 min), MeOH (2 × 2 min), CH_2_Cl_2_ (2 × 2 min), and DMF (6 × 1 min). Peptide elongation was carried out through sequential Fmoc removal and coupling steps of the corresponding Fmoc-protected amino acid. Fmoc group removal was performed with piperidine/DMF (3:7, 2 + 10 min). Couplings of the Fmoc-amino acids (4 equiv.) were mediated by ethyl 2-ciano-2-(hydroxyimino)acetate (Oxyma) (4 equiv.), and DIC (4 equiv.) in DMF at room temperature for 1 h under stirring. The completion of the couplings was checked using the Kaiser test [59]. After each coupling and deprotection step, the resin was washed with DMF (6 × 1 min) and CH_2_Cl_2_ (2 × 1 min). Once the peptidyl sequence was completed, the resin was treated with piperidine/*N*-methyl-2-pyrrolidinone (NMP) (3:7, 2 + 10 + 10 min), washed with NMP (6 × 1 min), CH_2_Cl_2_ (3 × 1 min), and diethyl ether (3 × 2 min), and air-dried. Finally, the resulting resin was treated with TFA/H_2_O/triisopropylsilane (TIS) (95:2.5:2.5) for 2 h at room temperature. Following TFA evaporation and diethyl ether extraction, the crude peptide was dissolved in H_2_O, lyophilized, purified with a CombiFlash, analyzed by HPLC, and characterized by ESI-MS and HRMS.

### 4.3. Bacterial Strains and Growth Conditions

The following plant pathogenic bacterial strains were used as target bacteria for in vitro experiments: *E. amylovora* PMV6076 (Institut National de la Recherche Agronomique, Angers, France) and EPS101 (Institut de Tecnologia Agroalimentària, Universitat de Girona, Spain), *P. syringae* pv. actinidiae Psa3700.1.1 (Instituto Valenciano de Investigaciones Agrarias, Valencia, Spain), *P. syringae* pv. syringae EPS94 (Institut de Tecnologia Agroalimentària, Universitat de Girona, Spain), *X. arboricola* pv. pruni CFBP5563 (Collection Française de Bactéries associées aux Plantes, Angers, France), *X. axonopodis* pv. vesicatoria 2133-2, and *X. fragariae* Xf349-9A (Instituto Valenciano de Investigaciones Agrarias, Valencia, Spain). All bacteria, except for *X. fragariae,* were stored in Luria Bertani (LB) broth supplemented with glycerol (20%) and maintained at −80 °C. For *X. fragariae*, Medium B [60] was used instead of LB. *E. amylovora*, *P. syringae* pv. actinidiae, *P. syringae* pv. syringae, and *X. arboricola* pv. pruni*,* were scraped from the agar media after growing for 24 h at 25 °C, and *X. axonopodis* pv. vesicatoria, and *X. fragariae* after growing for 48 h at 25 °C. The cell material was suspended in sterile water to obtain a suspension of 10^8^ CFU mL^−1^. *E. amylovora* EPS101 isolated from an infected shoot of a Conference pear tree in Lleida (Spain) was used for the in planta assays [61].

### 4.4. Antibacterial Activity

Lyophilized peptides were solubilized in sterile Milli-Q water to a final concentration of 1 mM and filter sterilized through a 0.22 m pore filter. Minimum inhibitory concentration (MIC) of the peptides was assessed as previously described [11] using a growth inhibition assay. Dilutions of the peptides were made to obtain a final concentration of 100, 50, 25, 12.5, 6.2, 3.1, and 1.6 μM, and in the case of the peptide mixture, an equimolar solution was used. Kanamycin sulfate (Sigma, St. Louis, MO, USA) was included for *E. amylovora* as a reference antibiotic. Twenty microlitres of each dilution were mixed in a microtiter plate well with 20 μL of the corresponding bacterial suspension (final concentration 10^7^ CFU mL^−1^) and 160 μL of Tryptic Soy Broth (TSB) (BioMèrieux, Marcy-l’Étoile, France) to a total volume of 200 μL. Three replicates for each peptide and concentration were used. Bacterial growth was determined by optical density measurement at 600 nm (Bioscreen C, Labsystem, Helsinki, Finland). Microplates were incubated at 25 °C with 20 s shaking before hourly absorbance measurement for 48 h. The experiment was repeated twice. The MIC was taken as the lowest peptide concentration with no growth at the end of the experiment.

### 4.5. Analysis of Bactericidal Activity

The bactericidal activity against *E. amylovora* was determined at the MIC (highest value observed in the growth inhibition assay) for the monomers **BP16** (50 μM) and **flg15** (100 μM), the conjugate **BP358** (6.2 μM), and an equimolar mixture of **BP16** and **flg15** (25 μM), and it was compared to an untreated control. TSB broth-grown cultures of *E. amylovora* inoculated at 10^7^ CFU ml^−1^ were incubated at the corresponding peptide or mixture concentration. Aliquots of 100 μL were removed at 30-min intervals for 3 h and diluted 10-fold, and the dilutions were plated on LB agar plates. The CFU were counted after a 48-h incubation at 25 °C. The experiment consisted of three replicates per treatment. Values were expressed as log CFU ml^−1^ during the experiment.

### 4.6. Hemolytic Activity

The hemolytic activity of the peptides was evaluated by determining hemoglobin release from erythrocyte suspensions of horse blood (5% *v*/*v*) (Oxoid) as previously described [11]. Blood was centrifuged at 6000 *g* for 5 min, washed three times with TRIS buffer (10 mM TRIS, 150 mM NaCl, pH 7.2), and diluted ten-fold. Peptides were solubilized in TRIS buffer. To test the hemolytic activity of the peptide mixture, an equimolar solution was used. Solubilized peptides were mixed with horse erythrocytes, and the final concentrations tested were 375, 250, 150, and 50 µM. The mixture was incubated under continuous shaking for 1 h at 37 °C. Then, the tubes were centrifuged at 3500*g* for 10 min, 80 μL aliquots of the supernatant transferred to 100-well microplates (Bioscreen), diluted with 80 μL water, and the absorbance measured at 540 nm (Bioscreen). Complete hemolysis was obtained by the addition of melittin at 100 µM (Sigma-Aldrich Corporation, Madrid, Spain). The percentage of hemolysis (*H*) was calculated using Equation (1):

*H* = 100 × [(*Op* − *Ob*)/(*Om* − *Ob*)] (1)

where *Op* is the density for a given peptide concentration, *Ob* is the buffer, and *Om* is the melittin positive control.

### 4.7. Effect of Peptide Infiltration on Tobacco Leaves

Peptides were evaluated for their effect upon infiltration on tobacco leaves as described previously [13]. Peptide solutions of 50, 150, and 250 µM were infiltrated (100 μL) into the mesophylls of fully expanded tobacco leaves. To test the effect of the peptide mixture, an equimolar solution was used. Six independent inoculations were carried out in a single leaf, and at least three independent inoculations were performed per peptide and concentration randomly distributed in different leaves and plants. Control infiltrations with water (negative control) or melittin (positive control) at the same molar concentration were performed. The appearance of symptoms on the leaves was followed for 48 h after infiltration and measured as a lesion diameter.

### 4.8. Effect of Peptide Treatment on Induction of Defense Gene Expression of Tomato Plants

Seeds of tomato cv. Rio Grande plants were sown in hydroponic seed plugs (rockwool), germinated and grown under controlled greenhouse conditions (25 ± 2 °C, 16 h light/15 ± 2 °C, 8 h dark, and 60% relative humidity (RH). Two-week-old seedlings (two cotyledons) were transplanted into rockwool plugs (7.5 × 7.5 × 6.5 cm, Gordan Iberica, Spain). The experimental design consisted of three replicates of three plants per treatment. After two weeks, tomato leaves were sprayed with aqueous solutions of the peptides **BP16**, **flg15**, and **BP358**, an equimolar mixture of **BP16** and **flg15,** at 125 µM, or with acybenzolar-*S*-methyl at 300 mg/L (Syngenta, Basel, Switzerland) until the run-off point. Water-sprayed plants were used as untreated controls. Twenty-four hours after product application, leaf samples were collected and processed to extract RNA for RT-qPCR assays. Plant material was ground to a fine powder in liquid nitrogen, adding 2 acid-washed glass beads (Sigma, 150 ± 600 μm) to the sample using the Tissuelyzer II system (Qiagen, Hilden, Germany). Total RNA was extracted from leaves using PureLink Plant RNA Reagent (Invitrogen, Life Technologies) according to the manufacturer’s manual. The RNA was solubilized in RNAse free water and was routinely subjected to DNAse treatment (Ambion^®^ Turbo DNA-free™, Invitrogen Life Technologies, Carlsbad, CA, USA) to remove any contaminant DNA. In each step, the RNA was quantified using a Nanodrop N-2000 spectrophotometer, and its integrity was verified by denaturing agarose gel electrophoresis. First-strand of complementary DNA (cDNA) was generated from leave RNA using reverse transcriptase (High Capacity cDNA Reverse Transcription Kit, Applied Biosystems, Foster City, CA, USA) according to the manufacturer’s manual.

To test gene defense induction in the treated tomato plants, a quantitative PCR (qPCR) assay was performed. qPCR was carried out in a fluorometric thermal cycler (qPCR Quant Studio 5, Applied Biosystems) by using a Mix SYBR^®^Green PCR Master Mix (Applied Biosystems, Foster City, CA, USA) as previously described [4]. Melting curve analysis was performed after each amplification to verify amplification specificity. A constitutive gene (*actin*) was used as a reference control, and the following genes implicated in plant defense response were analyzed: pathogenesis-related protein-1 (*PR1*), harpin (*Harp*), polyphenol oxidase (*PPO*), subtilisin-like protease (*Sub1*), blue copper binding-protein (*BCB*), osmotin (*Osm2*), acidic β-1,3 endoglucanase (*GluA*), lipoxygenase (*LOX*), protein inhibitor II (*PinII*), dehydrin (*Tas14*), and early-ripening tomato (*ERT3*). Specific oligonucleotides genes [4,55] were designed and used for their quantification. The primer concentration was 100 nM for all the genes except for the *GluA*, *Harp*, *PR1*, and actin genes which concentration was 300 nM. A calibration curve was prepared using decimal dilutions of recombinant plasmid DNA (target sequences were cloned into a vector pSpark (Canvax, Córdoba, Spain) in *Escherichia coli* DH5α cells). The efficiency for each standard curve was calculated to check that the efficiency within amplifications was similar.

Relative quantification of gene expression was done using the ΔΔ*Ct* method as previously described [4,52]. Ct values obtained for each repetition treatment were used to estimate the fold change value of the endogenous reference gene (actin) and the target plant defense genes. These results were used to calculate the ratios of the plant defense genes (relative to the actin gene and for all treatments analyzed, including the control plants). The statistical significance of the results for the selected peptides was determined using the REST2009 Software (Qiagen) [62].

### 4.9. In Planta Assays

The efficacy of peptides in fire blight suppression was determined using whole-plant infection assays with pear plants (*Pyrus communis* cv. Conference, Agromillora, Iberica S.A, Spain) inoculated with *E. amylovora*. Three-year-old pear plants grown in 20 cm diameter plastic pots in the greenhouse were used. During winter, plants were left outside the greenhouse for chilling, and during early spring were pruned to leave 3 or 4 shoots and were forced to bud in the greenhouse. Plants were fertilized once a week with a 200-ppm N/P/K solution (20:10:20) and were used when the shoots were about 3 or 4 cm long and had 5 or 6 young leaves per shoot. Pear plants were sprayed with a hand sprayer with 6 mL of an aqueous suspension of peptides **BP358**, **BP16**, or **flg15** or with an equimolar mixture of **BP16** and **flg15**. Peptides were assayed at 125 µM. After 48 h, treated plants were spray inoculated until the run-off point (6 mL) with a suspension of *E. amylovora* at 10^8^ CFU/mL mixed with diatomaceous earth at 1 mg/mL. Plants were incubated under controlled greenhouse conditions (25 ± 2 °C, 16 h light/15 ± 2 °C, 8 h dark, and 60% RH). The experimental design consisted of three replicates of three plants per treatment. Water-sprayed plants were used as untreated control and plants treated with kasugamycin 8% at 2 g/L (Lainco, Rubí, Spain) as a positive control. Two independent experiments were performed. Incidence of infections was determined per each replicate after 5 days of pathogen inoculation as the percentage of leaves with symptoms [63]. The effect of peptide treatments on plant material infection was determined using analysis of variance (ANOVA) with the general linear model (GLM) procedure of the statistical analysis system (SAS) (Version 8.2, SAS Institute, Cary, NC, USA). Means were separated using Duncan’s test (*p* < 0.05).

## 5. Conclusions

In conclusion, this manuscript shows that the conjugation or the mixture of the plant elicitor peptide **flg15** with the antimicrobial peptide **BP16** could be suitable approaches to fight plant diseases caused by *E. amylovora*. It is worth highlighting the peptide conjugate **BP358**, incorporating **BP16** at the C-terminus of **flg15**, that exhibited high activity in planta as well as the capacity to induce plant defense responses. In fact, to the best of our knowledge, this is the first example of a peptide conjugate with activity against plant pathogens resulting from the combination of two monomers with different biological activities. Remarkably, in the pear-*E. amylovora* pathosystem, **BP358** was as effective as the antibiotic kasugamycin, being a promising alternative tool to be included in plant disease management strategies.

## Figures and Tables

**Table 1:** Sequences, retention times, HPLC purities, and mass spectrometry data of the peptides.

Peptide	Sequence	*t*_R_ (min) ^1^	Purity (%) ^2^	HRMS
**BP16**	KKLFKKILKKL-NH_2_	6.04	>99	1386.0070 [M + H]^+^
**flg15**	RINSAKDDAAGLQIA-OH	5.80	>99	1542.8242 [M + H]^+^
**BP358** (flg15-BP16)	RINSAKDDAAGLQIA-KKLFKKILKKL-NH_2_	6.94	>99	1455.9087 [M + 2H]^2+^
**BP359** (BP16-flg15)	KKLFKKILKKL-RINSAKDDAAGLQIA-OH	6.04	>99	1456.4023 [M + 2H]^2+^

^1^ HPLC retention time. ^2^ Percentage determined by HPLC at 220 nm after purification by column chromatography.

**Table 2:** Antimicrobial activity (MIC), hemolysis, and phytotoxicity of the peptides.

Peptide	MIC (μM)						Hemolysis ^2^ (%)	Tobacco Lesion ^3^(cm)
	*Ea* ^1^	*Pss* ^1^	*Psa* ^1^	*Xap* ^1^	*Xf* ^1^	*Xav* ^1^	375 μM	250 μM
**BP16**	25–50	6.2–12.5	3.1–6.2	6.2–12.5	12.5–25	12.5–25	3 ± 3.5	0.26 ± 0.1
**flg15**	>100	>100	>100	>100	>100	>100	0 ± 0	0 ± 0
**BP358** (flg15-BP16)	3.1–6.2	6.2–12.5	3.1–6.2	3.1–6.2	1.6–3.1	1.6–3.1	5 ± 0.2	0.42 ± 0.1
**BP359** (BP16-flg15)	> 50	12.5–25	12.5–25	25–50	12.5–25	3.1–6.2	0.8 ± 0.5	0.30 ± 0.1
**BP16 + flg15** ^4^	12.5–25	6.2–12.5	3.1–6.2	3.1–6.2	25–50	12.5–25	1 ± 0.3	0.30 ± 0.1

^1^ Ea: Erwinia amylovora; Pss: Pseudomonas syringae pv. syringae; Psa: Pseudomonas syringae pv. actinidiae; Xap: Xanthomonas arboricola pv. pruni; Xf: Xanthomonas fragariae; Xav: Xanthomonas axonopodis pv. vesicatoria. ^2^ Percent hemolysis at 375 μM plus confidence interval (α = 0.05). ^3^ Phytotoxicity at 250 μM determined as the lesion diameter (cm) in infiltrated tobacco leaves plus confidence interval (α = 0.05). ^4^ The assays were performed using a mixture of these two peptides at equal molar quantities.

**Table 3:** Expression of genes related to defense response in tomato after the treatment with the reference compound acibenzolar-*S*-methyl (ASM), peptides **flg15**, **BP16**, and **BP358**, and a mixture of **BP16** and **flg15** (**BP16** + **flg15**). Fold induction above 2 was considered overexpression in the relative quantification using the ΔΔ*Ct* method. Significant values are indicated in bold.

	Reference ^1^	Peptides ^2,3^			
Genes	ASM	flg15	BP16	BP358	BP16 + flg15 ^3^
*Harp*	**3.8**	**3.3**	0.9	**4.6**	1.0
*PR1*	**2.8**	**30.4**	0.1	0.3	0.2
*GluA*	**4.9**	**12.7**	0.1	**4.2**	**2.8**
*PPO*	**5.6**	**4.8**	1.6	0.7	0.3
*LOX*	**18.7**	1.6	0.9	**2.3**	0.4
*PinII*	**2.6**	**5.2**	0.4	0.5	1.5
*Sub1*	**3.9**	**12.3**	0.3	**3.8**	1.4
*ERT3*	**3.9**	**3.6**	0.2	0.9	0.9
*BCB*	**3.5**	**11.3**	0.6	**6.1**	**3.5**
*Osm2*	1.5	**11.3**	0.7	**2.5**	0.5
*Tas14*	0.1	**2.3**	**2.0**	0.1	0.1

^1^ The reference compound ASM was tested at 300 mg/L. ^2^ Peptides were tested at 125 μM. ^3^ The assays were performed using a mixture of these two peptides at equal molar quantities. The following colour grading represent the relative quantification (ΔΔ*Ct*): 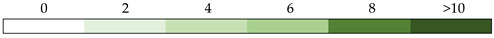

## Data Availability

Not applicable

## Supplementary Materials

The following are available online, HPLC, ESI-MS, and HRMS of peptides **BP16** and **flg15**, and of peptide conjugates **BP358** and **BP359**.

## Sample Availability

Samples of the compounds are available from the authors.
