# Supplementary material for: A Bifunctional Peptide Conjugate That Controls Infections of Erwinia amylovora in Pear Plants
Source: Molecules. 2021 Jun 5;26(11):3426. doi: 10.3390/molecules26113426 (PMC8201157; doi:10.3390/molecules26113426)
Supplement: Supplementary file 1 [file molecules-26-03426-s001.zip › molecules-1200569-SI.pdf]

Supporting Information

A bifunctional peptide conjugate that controls infections of *Erwinia amylovora* in pear plants

Pau Caravaca-Fuentes, Cristina Camó, Àngel Oliveras, Aina Baró, Jesús Francés, Esther Badosa, Marta Planas, Lidia Feliu, Emilio Montesinos and Anna Bonaterra*

Table of contents:

1. Characterization of peptides **BP16** and **flg15**: HPLC, ESI-MS and HRMS..............................2

2. Characterization of peptide conjugates **BP358** and **BP359**: HPLC, ESI-MS and HRMS.........8

1. Characterization of peptides BP16 and flg15

Lys-Lys-Leu-Phe-Lys-Lys-Ile-Leu-Lys-Lys-Leu-NH_2 (BP16)

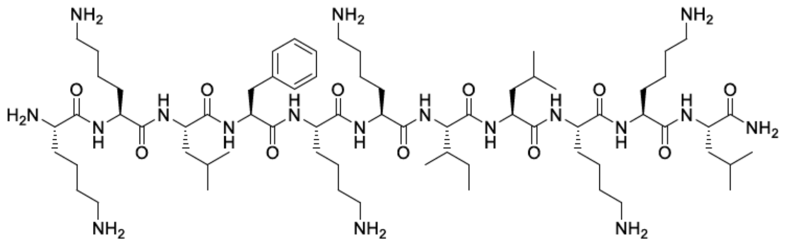

HPLC (λ=220 nm)

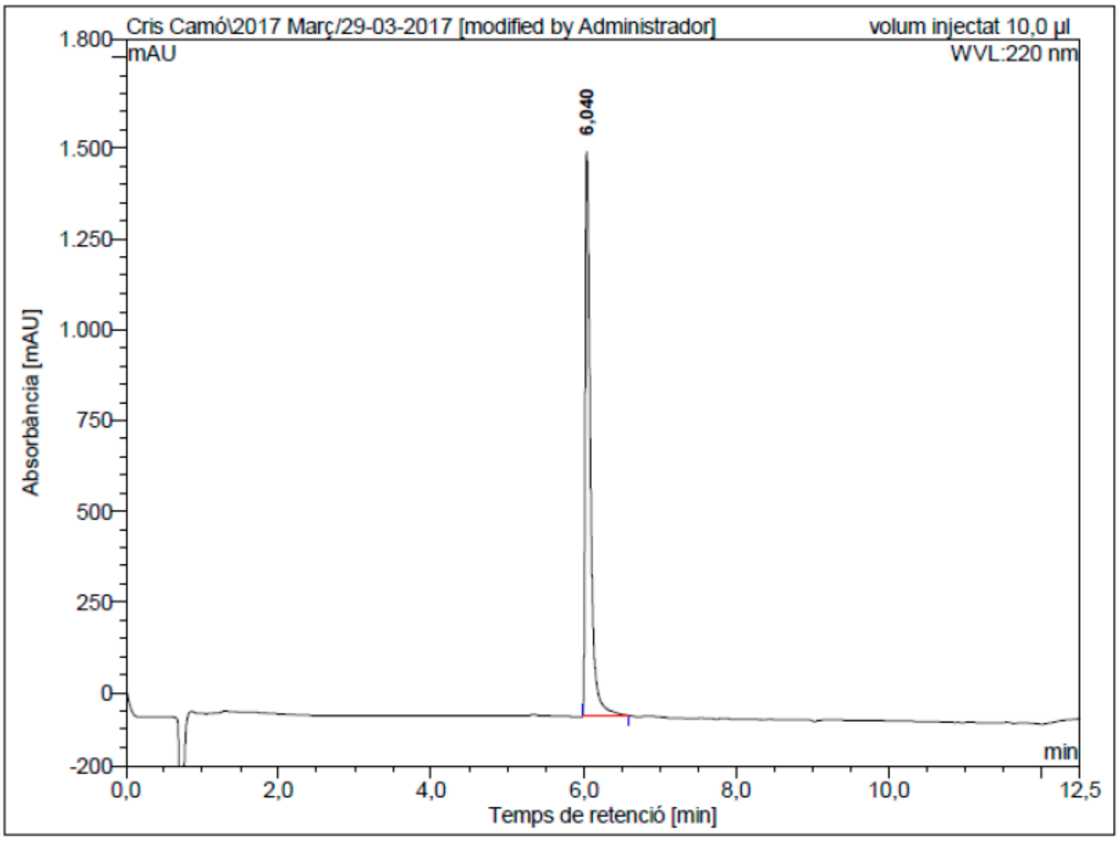

No.	Temps retenció min	alçada mAU	Area mAU*min	Area relativa %
1	6,04	1551,826	121,114	100,00
Total:		1551,826	121,114	100,00

ESI-MS (*m/z*)

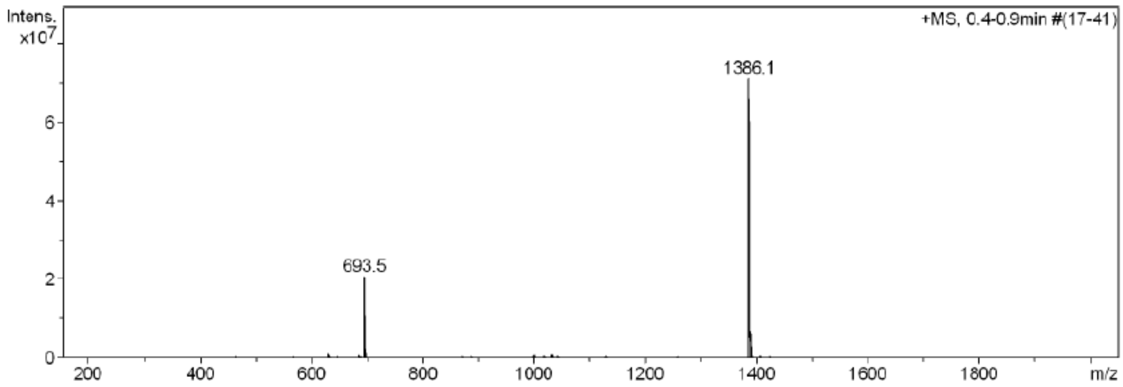

HRMS (*m/z*)

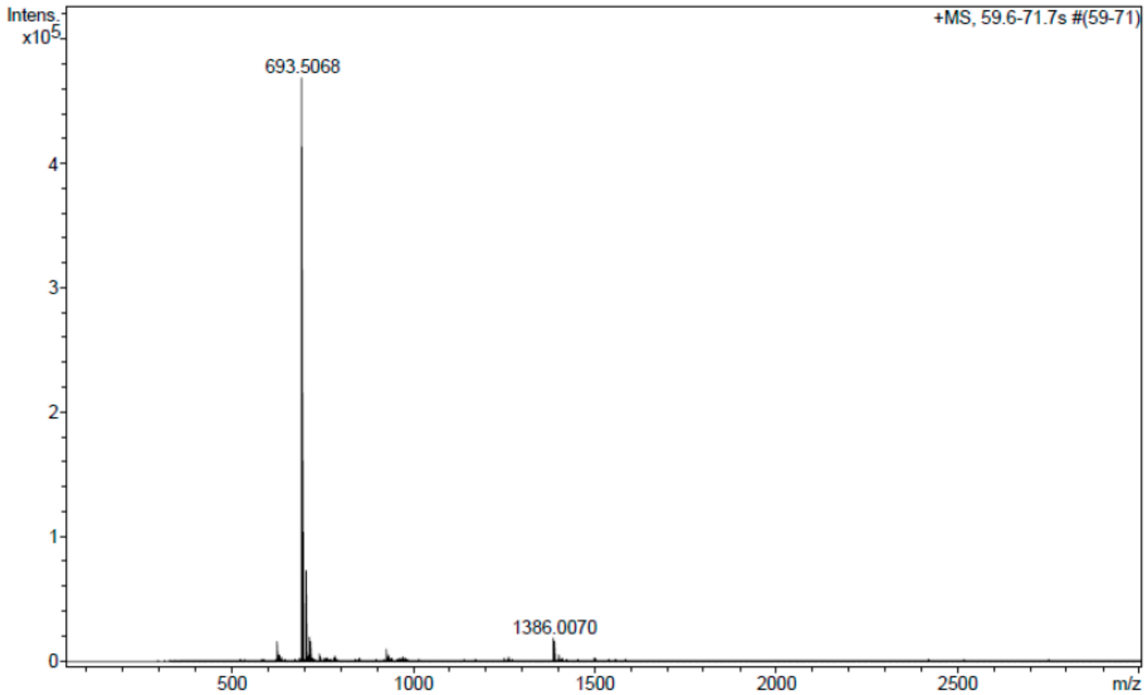

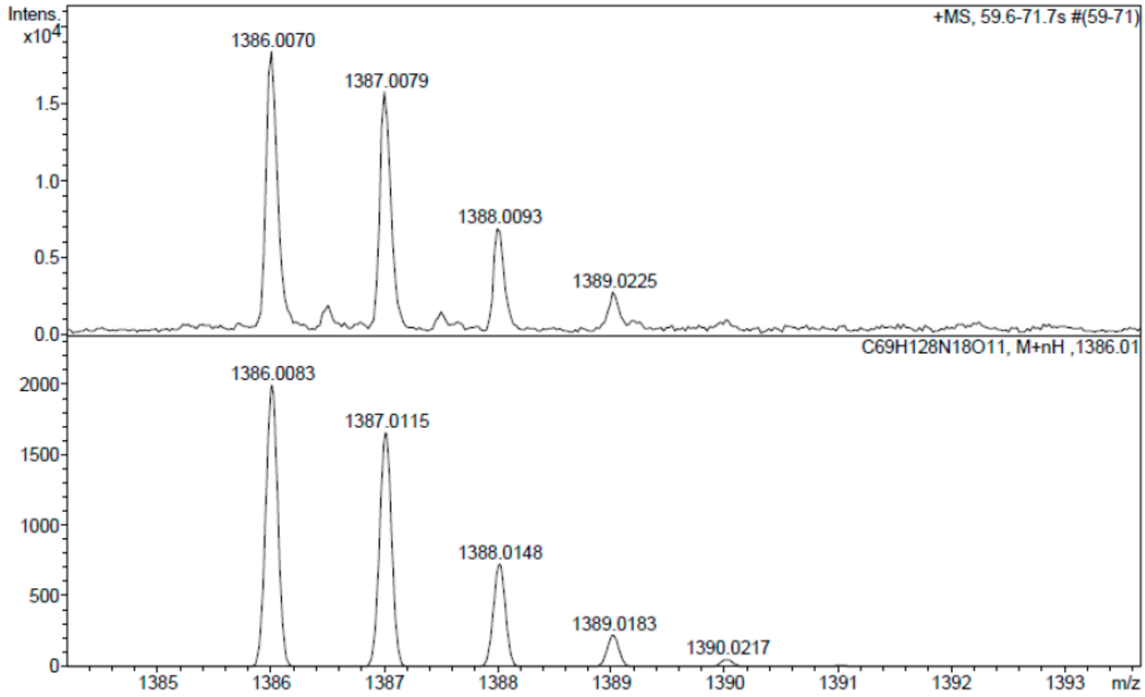
Intens.
x10^4
+MS, 59.6-71.7s #(59-71)
1386.0070
1387.0079
1388.0093
1389.0225
C69H128N18O11, M+nH ,1386.01
1386.0083
1387.0115
1388.0148
1389.0183
1390.0217
m/z

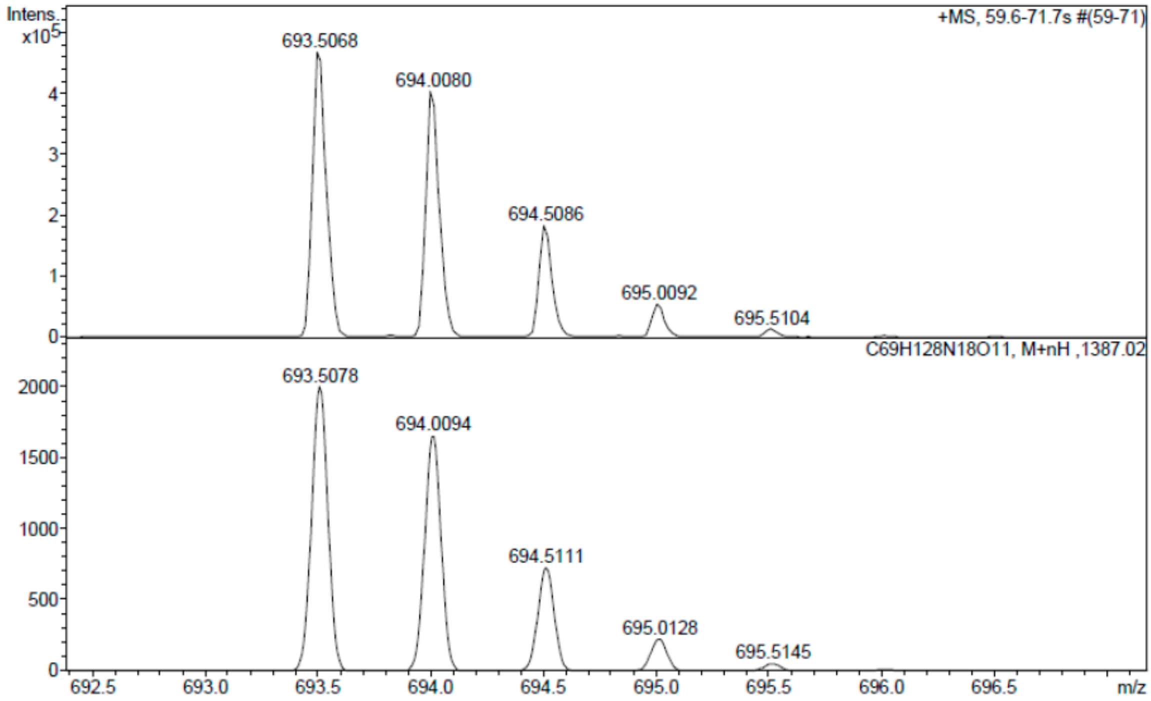
Intens.
x10^5
+MS, 59.6-71.7s #(59-71)
693.5068
694.0080
694.5086
695.0092
695.5104
C69H128N18O11, M+nH ,1387.02
693.5078
694.0094
694.5111
695.0128
695.5145
m/z

Arg-Ile-Asn-Ser-Ala-Lys-Asp-Asp-Ala-Ala-Gly-Leu-Gln-Ile-Ala-OH (flg15)

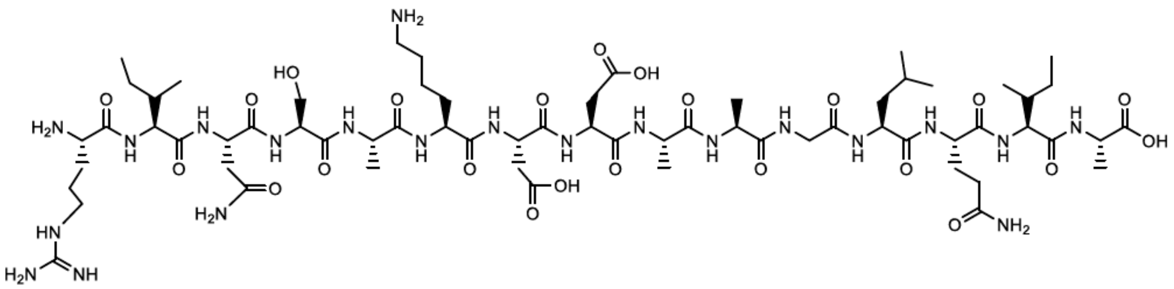

HPLC (λ=220 nm)

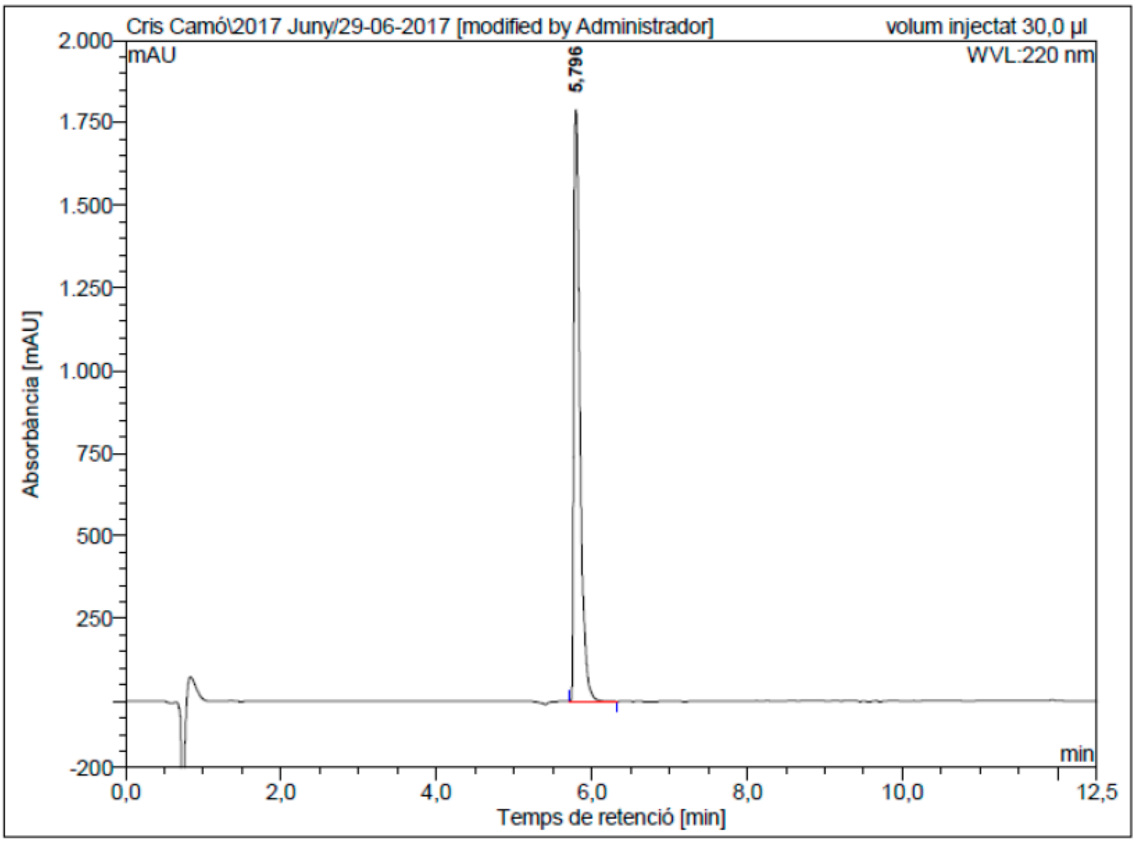

No.	Temps retenció<br>min	alçada<br>mAU	Area<br>mAU*min	Area relativa<br>%
1	5,80	1790,703	165,501	100,00
Total:		1790,703	165,501	100,00

ESI-MS (*m/z*)

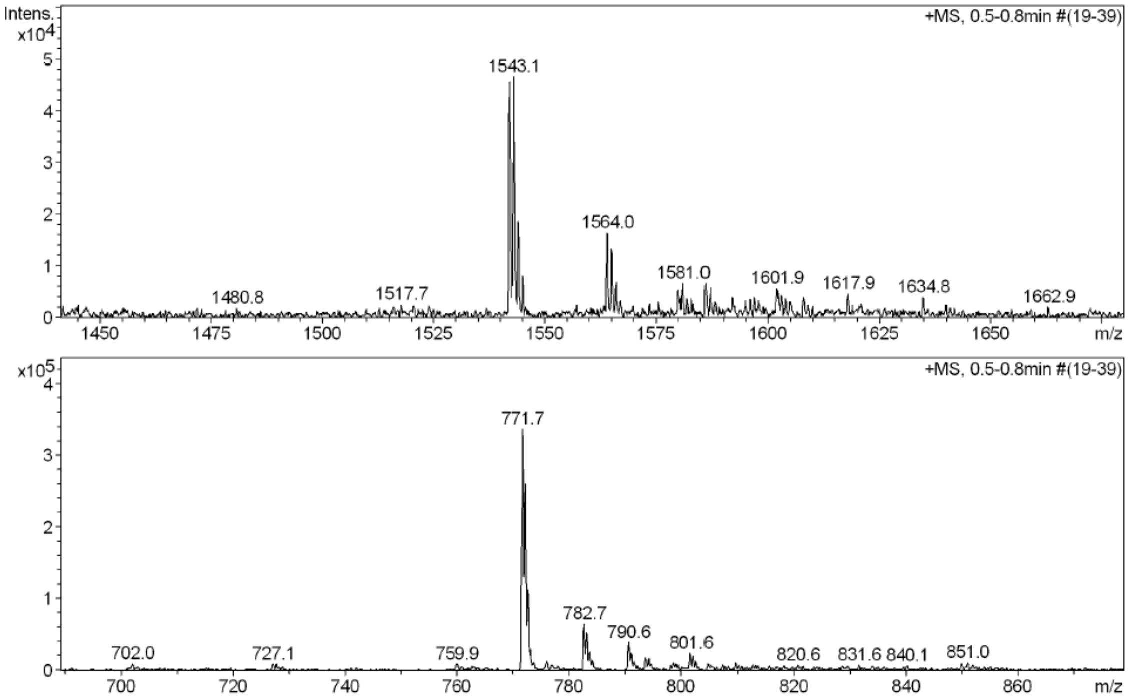

HRMS (*m/z*)

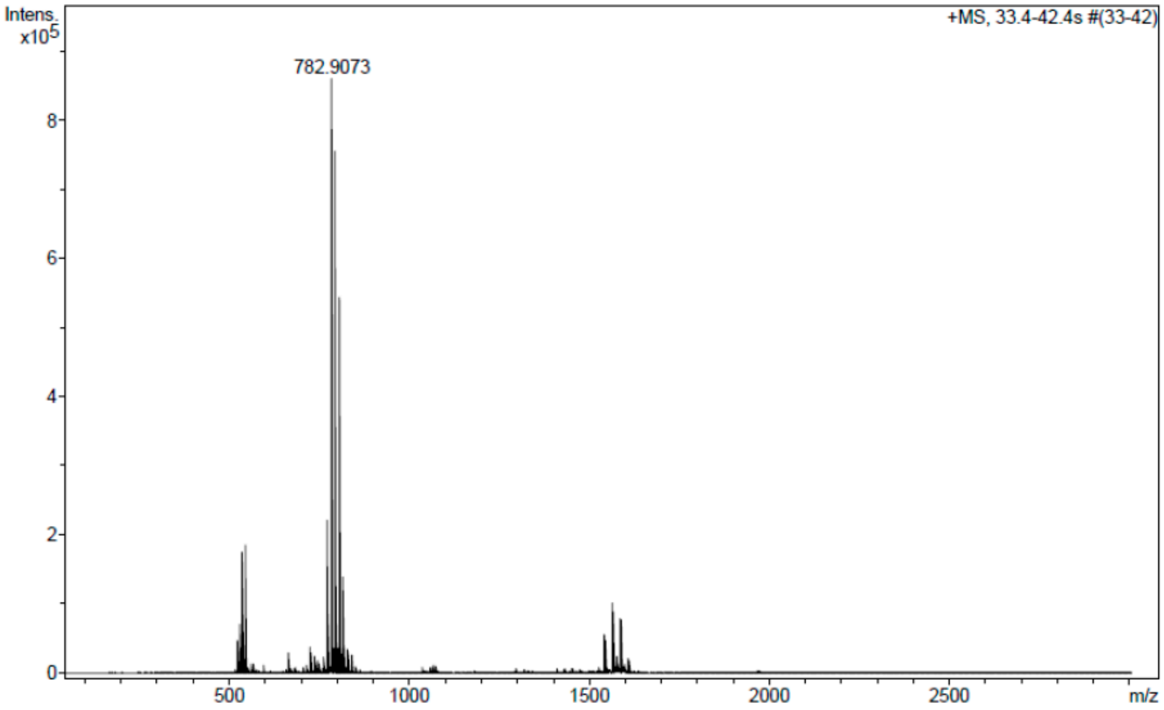

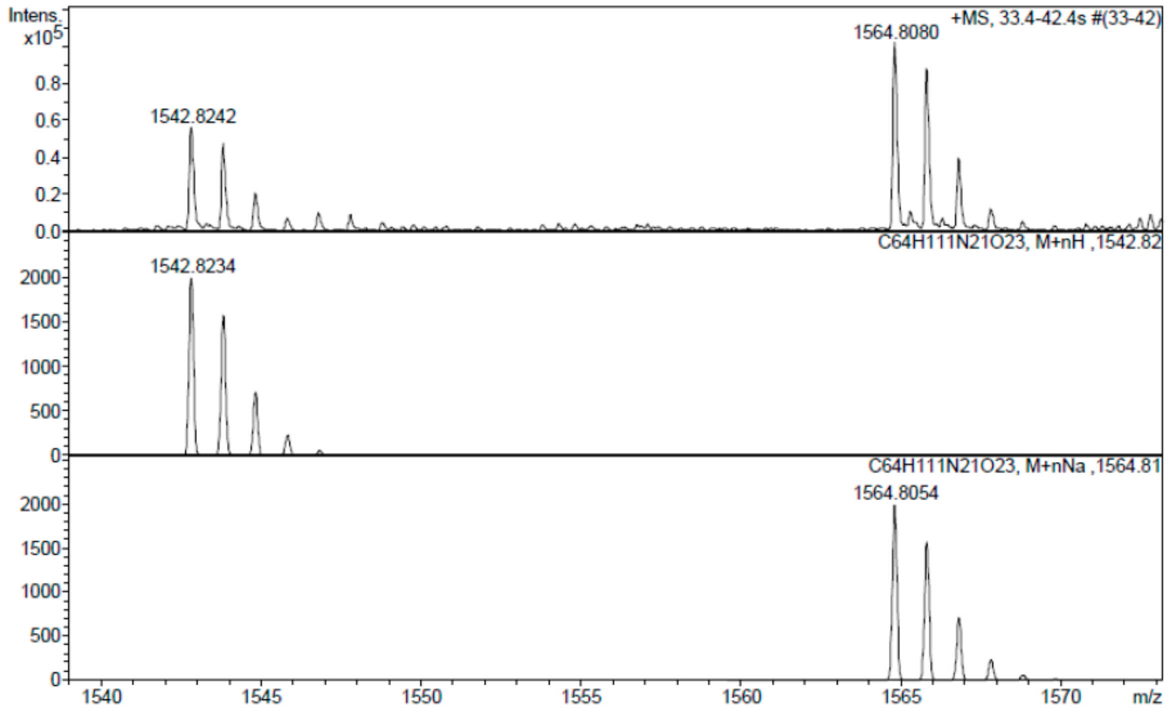
Intens.
x10^5
+MS, 33.4-42.4s #(33-42)
1564.8080
1542.8242
C64H111N21O23, M+nH ,1542.82
1542.8234
C64H111N21O23, M+nNa ,1564.81
1564.8054
m/z

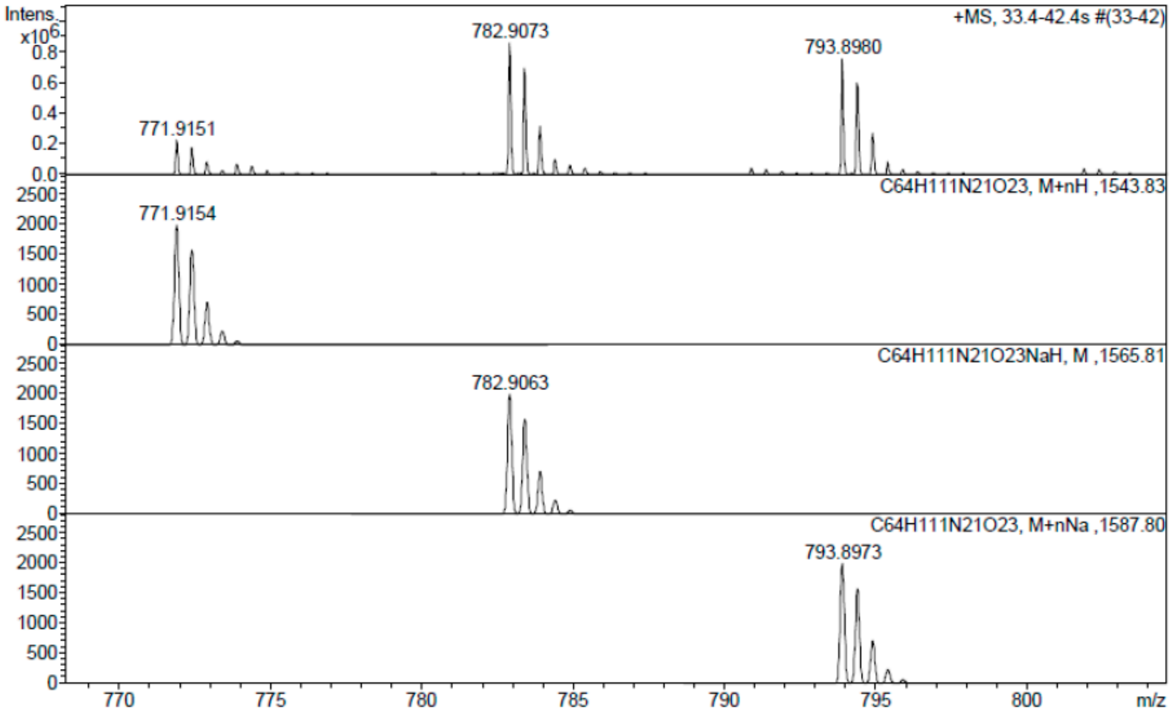
Intens.
x10^6
+MS, 33.4-42.4s #(33-42)
782.9073
793.8980
771.9151
C64H111N21O23, M+nH ,1543.83
771.9154
C64H111N21O23NaH, M ,1565.81
782.9063
C64H111N21O23, M+nNa ,1587.80
793.8973
m/z

2. Characterization of peptide conjugates BP358 and BP359

Arg-Ile-Asn-Ser-Ala-Lys-Asp-Asp-Ala-Ala-Gly-Leu-Gln-Ile-Ala-Lys-Lys-Leu-Phe-Lys-Lys-Ile-Leu-Lys-Lys-Leu-NH_2 (BP358)

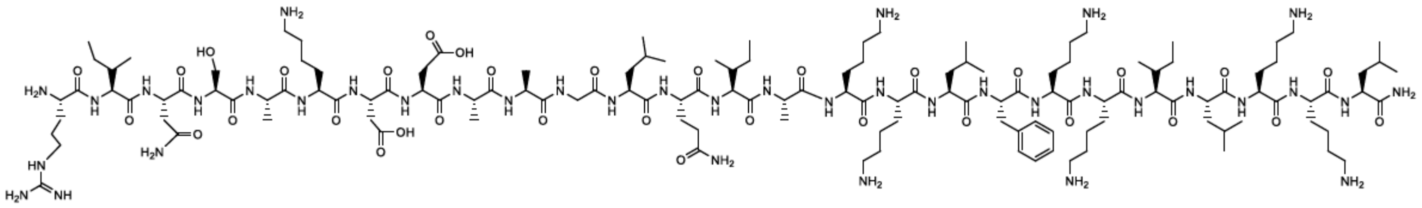

HPLC (λ=220 nm)

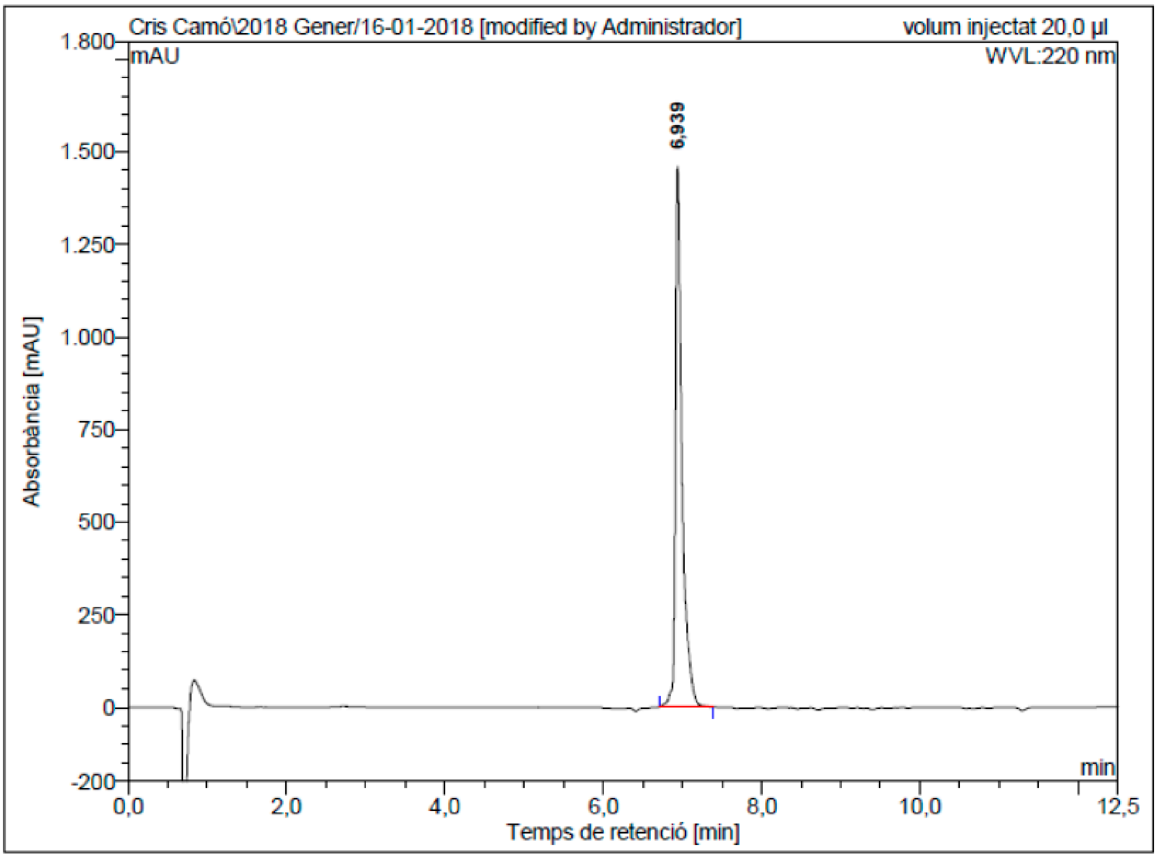

No.	Temps retenció min	alçada mAU	Area mAU*min	Area relativa %
1	6,94	1457,802	142,886	100,00
Total:		1457,802	142,886	100,00

ESI-MS (*m/z*)

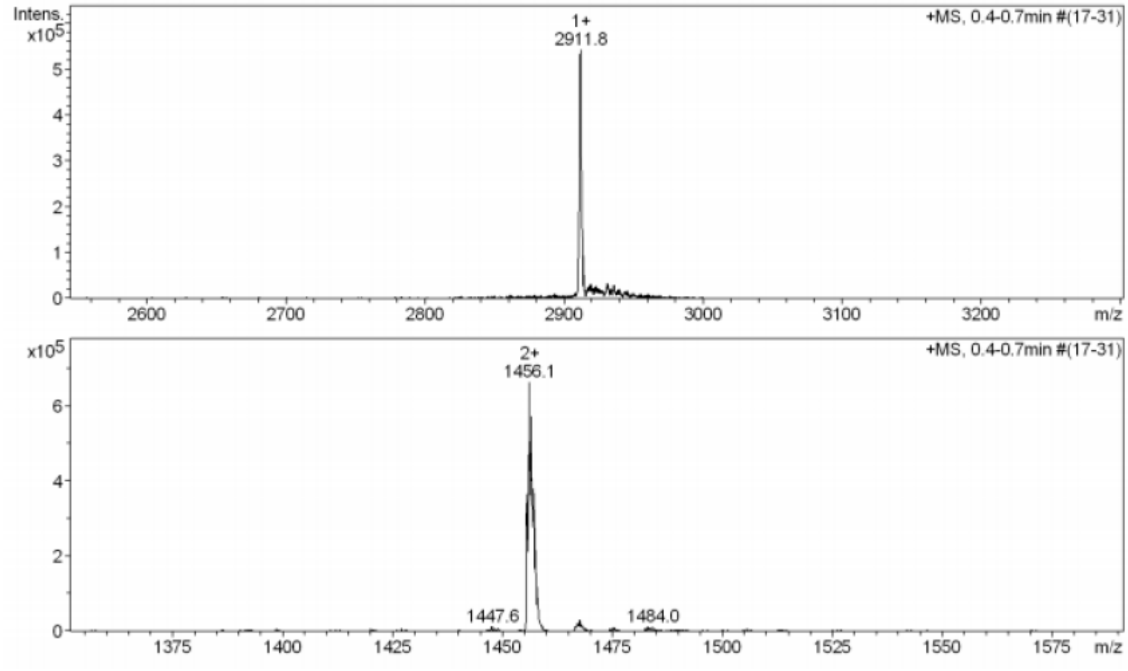

HRMS (*m/z*)

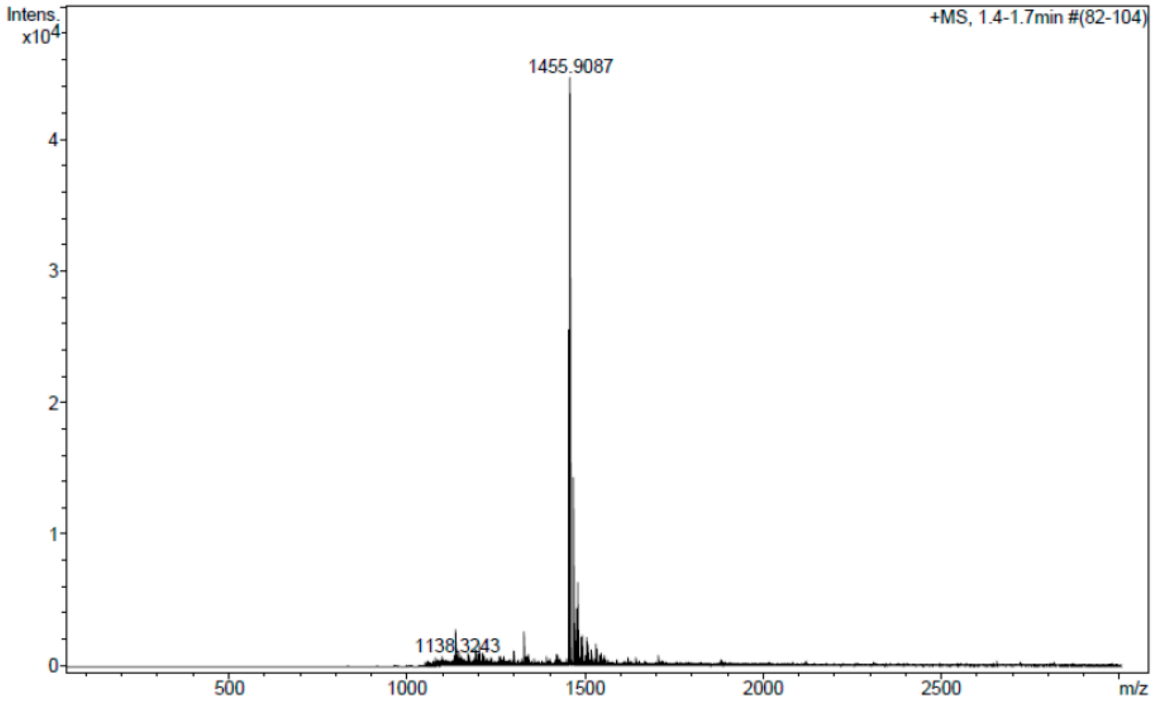

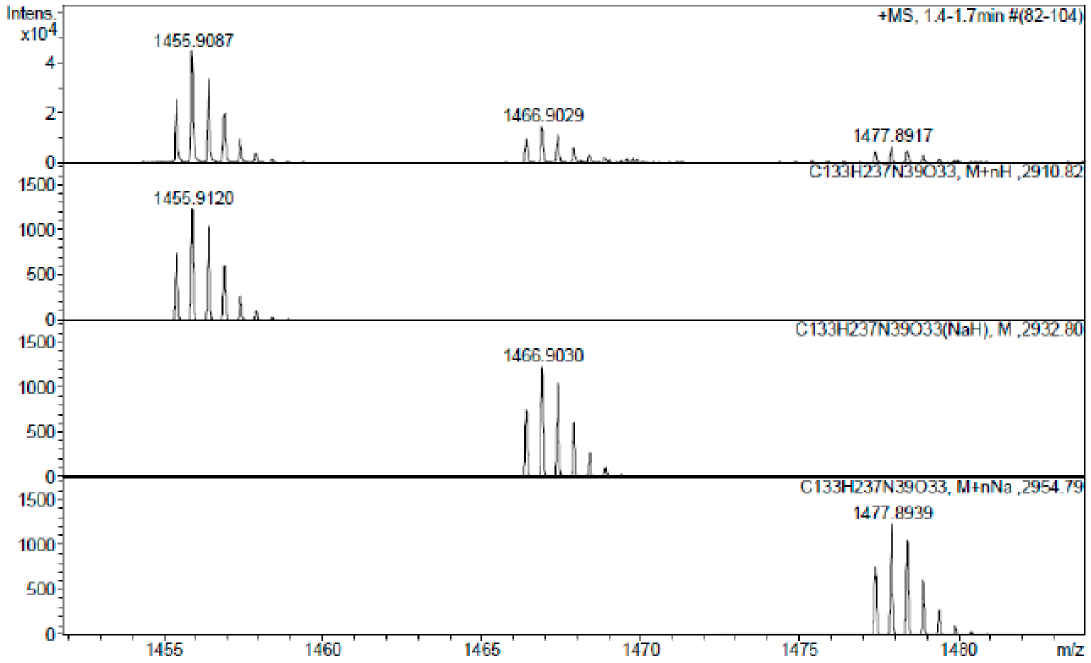
Intens.
x10^4
+MS, 1.4-1.7min #(82-104)
1455.9087
1466.9029
1477.8917
C133H237N39O33, M+nH ,2910.82
1455.9120
C133H237N39O33(NaH), M ,2932.80
1466.9030
C133H237N39O33, M+nNa ,2954.79
1477.8939
m/z

Lys-Lys-Leu-Phe-Lys-Lys-Ile-Leu-Lys-Lys-Leu-Arg-Ile-Asn-Ser-Ala-Lys-Asp-Asp-Ala-Ala-Gly-Leu-Gln-Ile-Ala-OH (BP359)

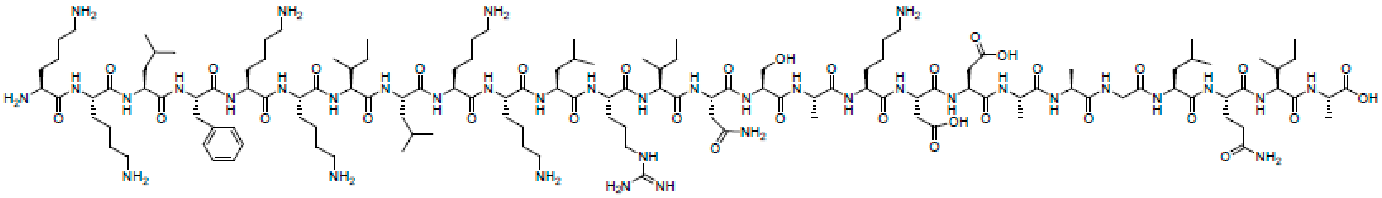

HPLC (λ=220 nm)

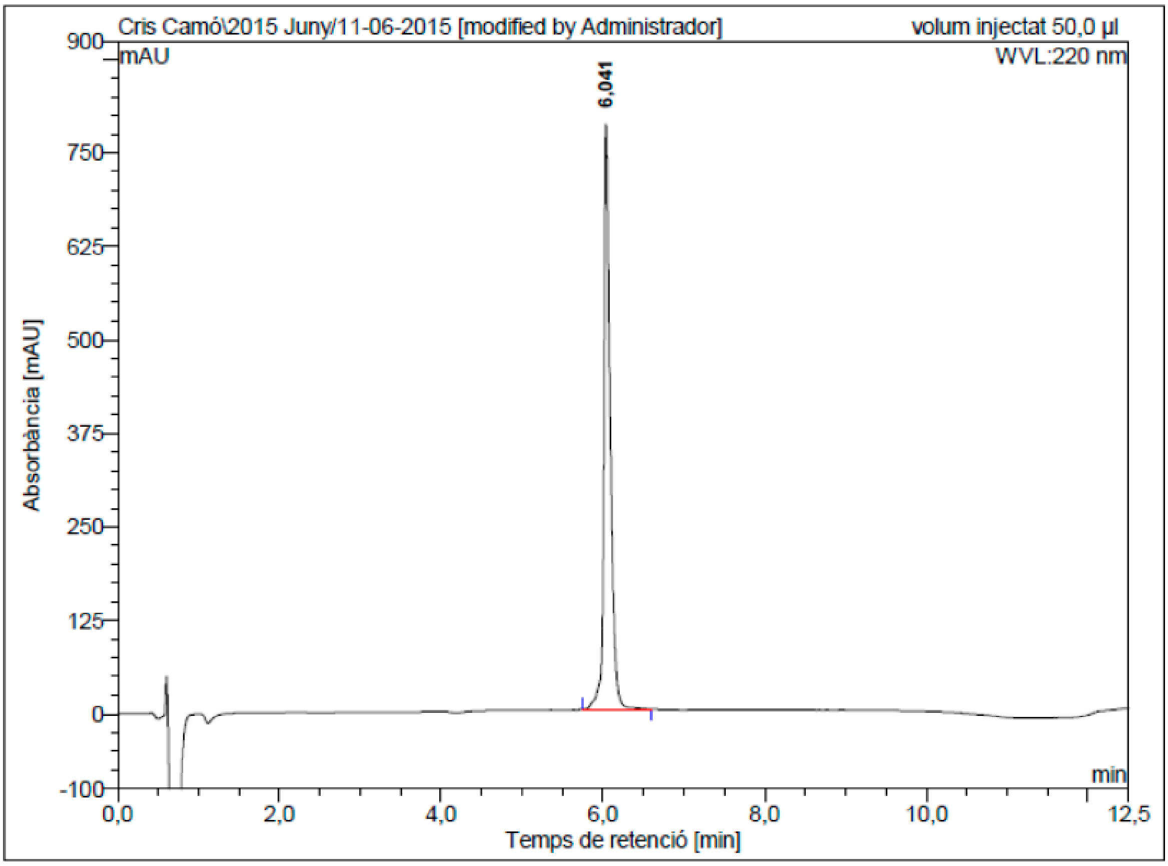

No.	Temps retenció min	alçada mAU	Area mAU*min	Area relativa %
1	6,04	782,370	71,301	100,00
Total:		782,370	71,301	100,00

ESI-MS (*m/z*)

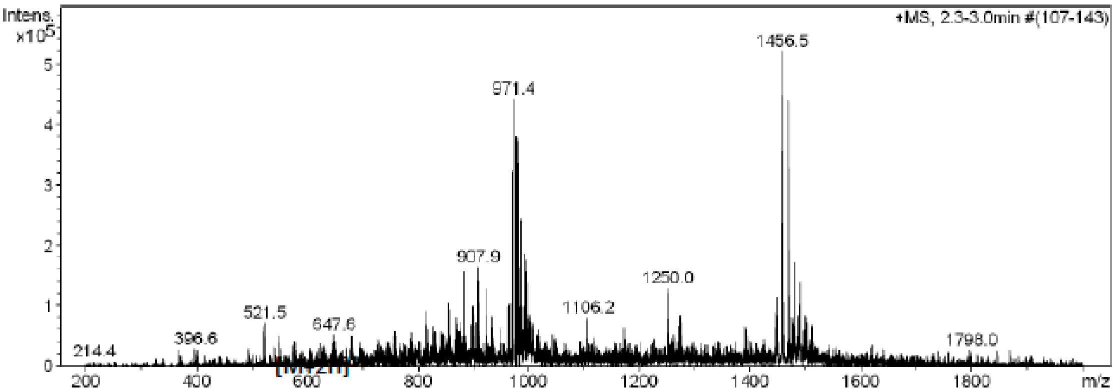

HRMS (*m/z*)

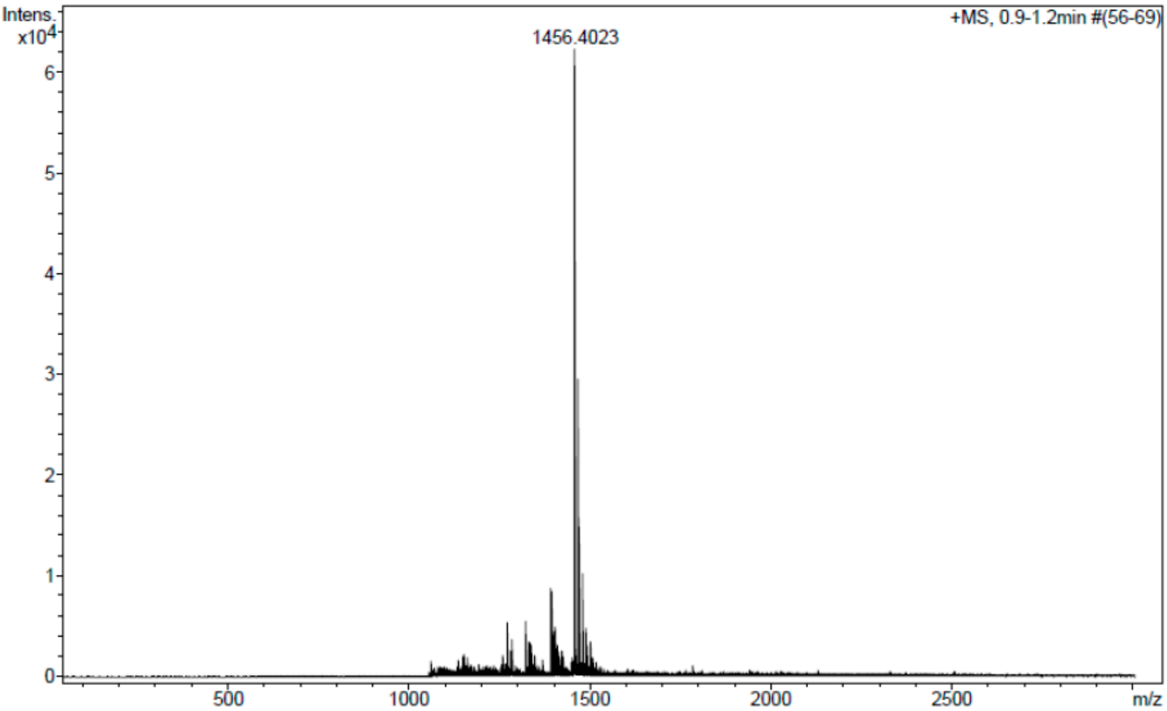

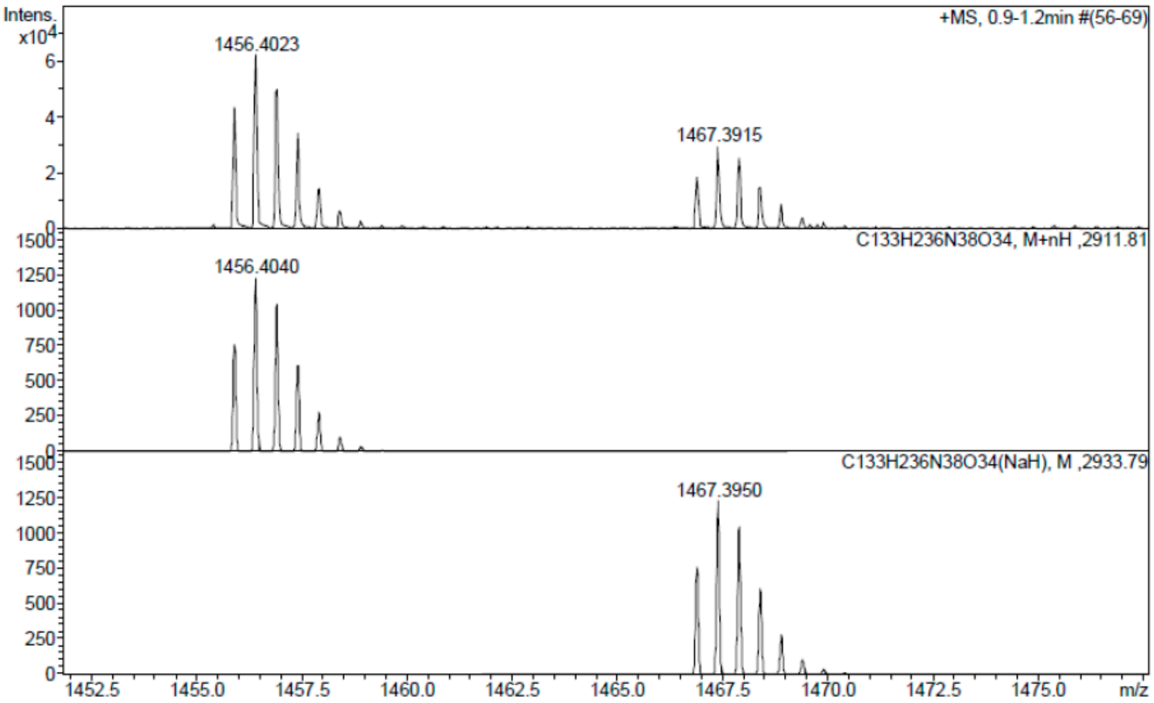
Intens.
x10^4
+MS, 0.9-1.2min #(56-69)
1456.4023
1467.3915
C133H236N38O34, M+nH ,2911.81
1456.4040
C133H236N38O34(NaH), M ,2933.79
1467.3950
m/z
